# EVIDENT 3 Study: A randomized, controlled clinical trial to reduce inactivity and caloric intake in sedentary and overweight or obese people using a smartphone application: Study protocol

**DOI:** 10.1097/MD.0000000000009633

**Published:** 2018-01-12

**Authors:** José I. Recio-Rodriguez, Manuel A. Gómez-Marcos, Cristina Agudo-Conde, Ignasi Ramirez, Natividad Gonzalez-Viejo, Amparo Gomez-Arranz, Fernando Salcedo-Aguilar, Emiliano Rodriguez-Sanchez, Rosario Alonso-Domínguez, Natalia Sánchez-Aguadero, Jesus Gonzalez-Sanchez, Luis Garcia-Ortiz

**Affiliations:** a Primary Health Care Research Unit, La Alamedilla Health Center, Health Service of Castilla y León (SACYL), Biomedical Research Institute of Salamanca (IBSAL), Spanish Network for Preventive Activities and Health Promotion (REDIAPP); b Department of Nursing and Physiotherapy; c Department of Medicine, University of Salamanca; d Centro de Salud Sta Ponça de Palma de Mallorca, Spanish Network for Preventive Activities and Health Promotion (REDIAPP); e Torre Ramona Health Center, Aragon Health Service, Zaragoza; f Casa del Barco Health Center, Castilla y León Health Service, Valladolid; g Cuenca III Health Center, Castilla la Mancha Health Service (SESCAM); h Department of Nursing, University of Extremadura; i Department of Biomedical and Diagnostic Sciences, University of Salamanca,Spain.

**Keywords:** arterial aging, food, mHealth, obesity, physical activity, weight loss

## Abstract

**Introduction:**

Mobile technology, when included within multicomponent interventions, could contribute to more effective weight loss. The objective of this project is to assess the impact of adding the use of the EVIDENT 3 application, designed to promote healthy living habits, to traditional modification strategies employed for weight loss. Other targeted behaviors (walking, caloric-intake, sitting time) and outcomes (quality of life, inflammatory markers, measurements of arterial aging) will also be evaluated.

**Methods:**

Randomized, multicentre clinical trial with 2 parallel groups. The study will be conducted in the primary care setting and will include 700 subjects 20 to 65 years, with a body mass index (27.5–40 kg/m^2^), who are clinically classified as sedentary. The primary outcome will be weight loss. Secondary outcomes will include change in walking (steps/d), sitting time (min/wk), caloric intake (kcal/d), quality of life, arterial aging (augmentation index), and pro-inflammatory marker levels. Outcomes will be measured at baseline, after 3 months, and after 1 year. Participants will be randomly assigned to either the intervention group (IG) or the control group (CG). Both groups will receive the traditional primary care lifestyle counseling prior to randomization. The subjects in the IG will be lent a smartphone and a smartband for a 3-month period, corresponding to the length of the intervention. The EVIDENT 3 application integrates the information collected by the smartband on physical activity and the self-reported information by participants on daily food intake. Using this information, the application generates recommendations and personalized goals for weight loss.

**Discussion:**

There is a great diversity in the applications used obtaining different results on lifestyle improvement and weight loss. The populations studied are not homogeneous and generate different results. The results of this study will help our understanding of the efficacy of new technologies, combined with traditional counseling, towards reducing obesity and enabling healthier lifestyles.

**Ethics and dissemination:**

The study was approved by the Clinical Research Ethics Committee of the Health Area of Salamanca (“CREC of Health Area of Salamanca”) on April 2016. A SPIRIT checklist is available for this protocol. The trial was registered in ClinicalTrials.gov provided by the US National Library of Medicine-number NCT03175614.

## Abstract 2

Supplemental Digital Content is available in the text

## 1 Introduction

### 1.1 The obesity problem

Obesity is growing epidemic in developed countries characterized by elevated body mass index (BMI) and associated with numerous health problems including cardiovascular issues (e.g., stroke, coronary disease, hypertension, type 2 diabetes mellitus).^[1,2]^ Obesity exacerbates primary cardiovascular risk factors (lipids, blood pressure, glucose, and inflammation)^[3]^ and contributes 20% of all mortalities^[4]^ and 40% of mortalities due to cardiovascular disease.^[5]^

The rapid increase in the prevalence of obesity, and its high disease burden, strengthens the need to identify, implement, and evaluate evidence-based interventions that address this problem.^[6]^ The existing evidence does not support the use of low- or moderate-intensity counselling alone in the treatment of obesity.^[7]^ Whereas complex, multipronged interventions have resulted in clinically significant weight loss (over 5%) in overweight and obese adults.^[8]^ Towards that end, the US Preventive Services Task Force recommends these intensive interventions partially because more patient contact in the first year results in greater, more sustainable weight loss.^[9]^

### 1.2 Lifestyle and obesity

A healthy lifestyle has been associated with a lower prevalence of obesity.^[10]^ The positive effects of regular physical activity on health and mortality are well established. Physical activity plays an important role in the prevention of various chronic diseases, such as cardiovascular disease, obesity, diabetes mellitus, osteoporosis, and colon cancer.^[11]^ However, in Europe, more than half of the adult population does not achieve the recommended levels of daily physical activity.^[12]^ Furthermore, sedentarism, defined as low levels of energy expenditure (≤1.5 MET basal metabolic rate), is related to multiple health problems: higher risk of diabetes, cardiovascular disease, cancer, and all causes of death.^[13]^ Generally, the most obesity-inducing lifestyle choice is unhealthy eating habits. Diet has been directly associated with the prevalence and development of obesity. In this regard, the Western diet, characterized by high energy density, high fat content, and low consumption of fiber, is associated with higher rates of obesity,^[14]^ including higher plasma glucose levels and an unfavorable lipid profile.^[15]^ On the other hand, the Mediterranean diet is correlated with lower rates of obesity in a population with high cardiovascular risk, which results in reduced insulin resistance and less inflammation.^[16]^

### 1.3 Obesity and arterial aging

Obesity is associated with arterial health.^[17]^ In the EVIDENT study, sedentarism, low physical activity, and certain nutritional patterns were found to correlate with hemodynamic measures of arterial aging, such as the peripheral augmentation index (PAIx).^[18–20]^ There are numerous devices available for evaluating central hemodynamics,^[21]^ including a wrist-worn tonometry device developed by Microsoft Research^[22]^ that are both easy and convenient to use.

### 1.4 Mobile interventions for improving lifestyle and weight loss

The use of smartphone applications applied to the field of medicine is in progressive increase.^[23]^ mHealth includes the use of mobile phones, patient monitoring devices, and other wireless systems that can be used in the prevention, diagnosis, and treatment of chronic diseases including but not limited to type 2 diabetes and hypertension^[24–26]^ in addition to promoting a healthy lifestyle.^[27]^ A recent meta-analysis showed that mobile technology tools can result in a mean reduction of 41 minutes per day of sitting time.^[28]^ The potential of these applications to change lifestyles in the population is very promising, but long-term studies are needed to evaluate their efficacy over time.^[29]^ A review of the topic quantified the effect of these interventions as a decrease of on average 1.44 kg in body weight and of 0.24 units on the BMI.^[30]^ These values are very similar to those shown in the review by Flores Mateo et al.^[31]^ Taken together, these results indicate that mobile interventions decrease adiposity (Evidence Class IA)^[29]^ with an efficacy dependent on and proportional to the frequency with which they are used.^[32]^

### 1.5 Weight loss and quality of life

Interventions that aim to reduce obesity in adults through a combination of diet and physical exercise have achieved improvements in quality of life,^[33]^ with sustainable long-term effects (24 months). However, according to a recent meta-analysis, these improvements were achieved primarily on a physical level, not a mental one.^[34]^

In the previous study (EVIDENT 2), an improvement was obtained in terms of increased self-reported physical activity and an attributable effect in improving dietary habits in general population,^[35,36]^ using a smartphone application. In this study protocol we present the third phase of the EVIDENT study where the following developed version of the application will be used in a population of special vulnerability due to its increased cardiovascular risk, obese, or overweight people.

The objective of this research is to assess the impact of the EVIDENT 3 application on weight loss when combined with traditional primary care lifestyle modifications in overweight or obese subjects. Secondary outcomes include changes in physical activity, sedentarism, caloric intake, quality of life, arterial aging, and pro-inflammatory markers.

## 2 Methods

### 2.1 Design and setting

This is a randomized, multicentre clinical trial with 2 parallel groups. The study will be conducted in a primary care setting. The Research Unit group from La Alamedilla Health Center, Biomedical Research Institute of Salamanca (IBSAL), will coordinate the project on 5 sites from the Network for Preventive Activity and Health Promotion (REDIAPP) (Salamanca, Valladolid, Cuenca, Palma de Mallorca, and Zaragoza).

### 2.2 Study population

All current patients of this 5 health centers will be eligible for this study. Once inclusion/exclusion criteria are met (detailed below), a healthcare professional will provide study subjects with oral and written information about the study. Informed consent will be obtained prior to any trial proceedings.

Inclusion criteria: subjects 20 to 65 years of age, BMI ≥27.5 and <40 kg/m^2^, classified based on sedentarism (<3 20 minutes sessions of vigorous intensity physical activity per week; ≥5 30 minutes sessions of moderate intensity physical activity [including walking] per week; or ≥5 sessions of any combination of moderate and vigorous intensity physical activity).^[37]^

Exclusion criteria: subjects with any of the following conditions will be excluded: musculoskeletal disease restricting walking; low-calorie diet prescribed and/or controlled by a healthcare professional or other weight loss program; use of other applications with the objective to lose weight; bariatric surgery in the past or planned in the next 12 months; treatment with any pharmaceutical, natural, or homeopathic formulation for weight loss; personal history of NYHA Grade II or higher heart failure^[38]^; type 2 diabetes; oncological disease actively being treated; terminal status; pregnancy; people living in the same household as another study participant; other causes at the investigator's discretion that prevent inclusion.

### 2.3 Sample size

The sample size calculation was performed for the primary study endpoint of weight loss. Accepting an α risk of 0.05 and a β risk of 0.20, with an standard deviation (SD) of 12 kg, estimated in subjects from EVIDENT 2 study,^[35]^ 592 subjects would be needed (296 per group) to detect a decrease in weight of ≥3 kg^[39]^ in the intervention group (IG) versus the control group (CG), taking into consideration a 15% loss to follow-up. This effect size represents a 3% to 5% difference between groups, which should produce clinically relevant health benefits.^[40]^ To minimize dropouts, a reminder of the next visit will be provided at the end of each visit, as well as 2 phone calls 15 and 5 days prior.

### 2.4 Randomization

Participants will be randomly assigned to either the IG or the CG. Randomization will be performed after informed consent is obtained. The allocation sequence will be generated through a standardized computer programme (Epidat 4.2)^[41]^ by an independent researcher and concealed until the trial arms are assigned.

### 2.5 Procedures

**Figure 1:**
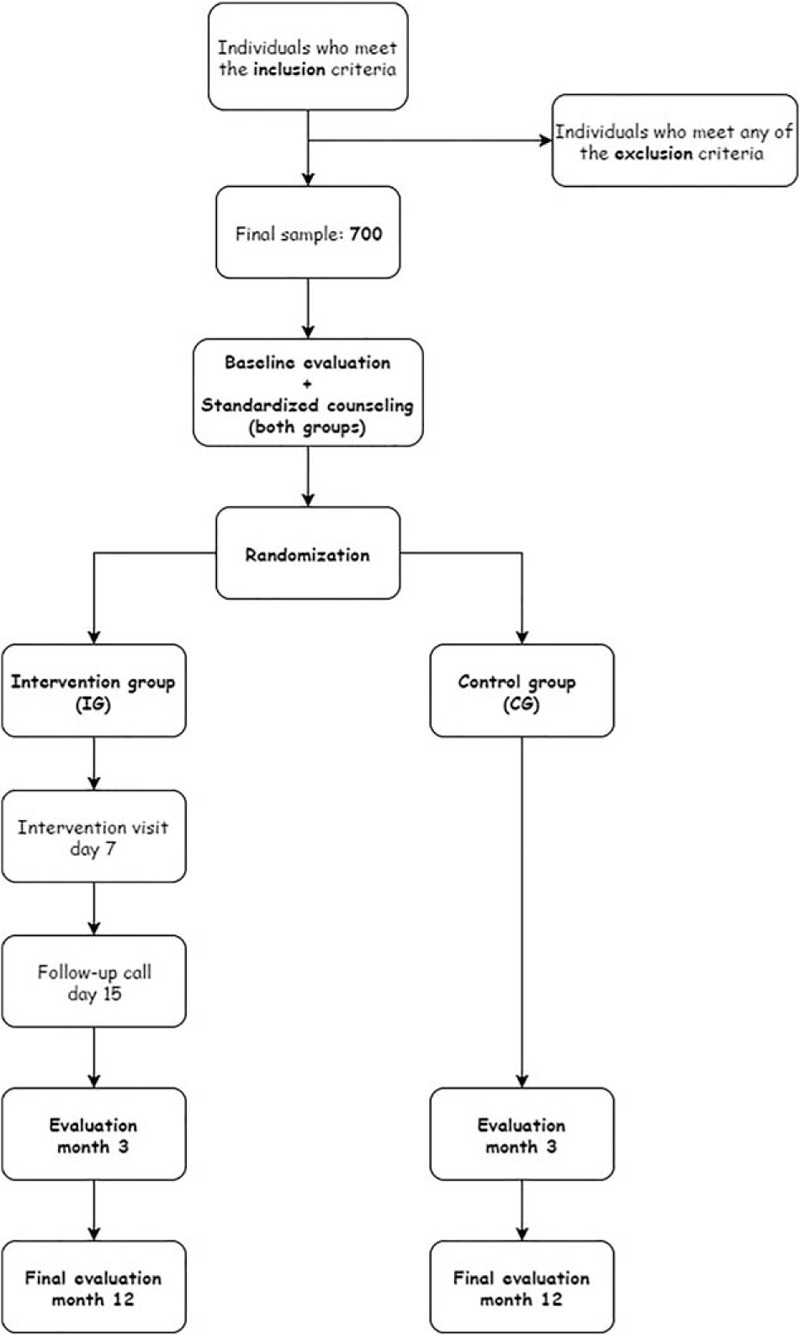
Study flowchart.

Each participant will complete an initial visit and 2 follow-up visits at 3 and 12 months after study initiation (Fig. 1). Baseline data will be collected by a research nurse. The IG will complete 2 additional appointments with a different nurse performing the measurements. The first one will occur 7 days from the baseline in which the application will be explained and the second one will be during a phone call 15 days from the baseline visit to answer any questions on how to use the application.

### 2.6 Primary and secondary outcomes

The primary outcome will be weight loss (kg). Secondary outcomes will include change in the physical activity (steps/d) measured by accelerometer, sitting time (min/wk), caloric intake (kcal/d), quality of life, arterial aging, and pro-inflammatory markers. All outcomes will be measured at baseline, 3, and 12 months after randomization.

#### 2.6.1 Weight loss

Body weight will be measured twice, with the subject barefoot and wearing light clothing, using a homologated electronic balance (Scale 7830; Soehnle Professional GmbH & Co, Backnang, Germany) after proper calibration (precision ± 0.1 kg). Height will be measured with the subject barefoot in the standing position to record the average of 2 readings rounded to the nearest centimeter, using a portable system (Seca 222; Medical scale and measurement system, Birmingham, UK). BMI will be calculated by weight (kg) divided by height squared (m^2^). Following the recommendations of the Spanish Society for the Study of Obesity (SEEDO),^[42]^ waist circumference will be measured in duplicate, using a flexible tape parallel to the floor, at the level of the midpoint between the last rib and the iliac crest, with the subject standing without clothing, after inspiration. Hip circumference will be similarly measured at the level of the trochanters. Body composition will be determined using an Inbody 230 analyzer^[43]^ that estimates total body water, dry lean mass, body fat mass, skeletal muscle mass, body fat percentage, distribution of lean body mass, ratio of segmental lean mass, and impedance of each body segment.

#### 2.6.2 Physical activity

Physical activity will be measured using both objective and subjective methods. Data from both self-reported and objective methods will be analyzed and published in study-derived manuscripts.

The primary means for quantifying physical activity objectively will be the number of steps/d measured by an Actigraph GT3X accelerometer (ActiGraph, Shalimar, FL), which has been previously validated.^[44]^ Output from the ActiGraph is in the form of activity “counts,” where 1 count is equivalent to 16 mg per second. Activity “counts” are recorded to the internal memory of accelerometers by converting acceleration units over a given period.^[45]^ The change in the quantity and intensity of the physical activity expressed in counts per minute, time dedicated to low, moderate, and high/very vigorous activity will be determined. All subjects included will wear the accelerometer on the right side of the waist, with an elastic band, for 7 consecutive days. Data will be recorded at 1-minute intervals. The intensity of the physical activity (low, moderate, or high) will be determined according to the cut-off points proposed by Freedson et al,^[46]^ sedentary activity (<100 counts/min), light (100–1952 counts/min), moderate (1952–5724 counts/min), vigorous (5724–9498 counts/min), and very vigorous (>9498 counts/min). The amount of physical exercise will be estimated in MET/h/wk, and active persons will be engaging in at least 30 minutes of moderate activity 5 days per week, or at least 20 minutes of vigorous activity 3 days per week, or who reach 450 MET/min/wk.^[47]^ Criteria for considering an accelerometer session to be valid include a minimum of 5 days, including at least 1 day of the weekend. Sequences of ≥10 consecutive zeros will indicate that the participant has not worn the device and will not be taken into account in the analysis.

The short version of the International Physical Activity Questionnaire (IPAQ)^[48]^ will be used to measure activity subjectively. The IPAQ is a self-reported questionnaire that evaluates sitting and active time in the last 7 days differentiating between walking, moderate-intensity, and vigorous-intensity activities, according to the energy expenditure estimated for each of them (3.3, 4.0, and 8.0 metabolic equivalents of task [MET], respectively). It allows the MET-min/wk to be calculated and subjects to be classified according to 3 activity levels (low, intermediate, and high).

#### 2.6.3 Sedentary lifestyle

The sitting time (min/wk) will be assessed using the ActivPal device. ActivPAL has been proven to be a practical, reliable, and valid instrument for objectively recording sedentary activity.^[49]^ This device will be adhered to the front of the thigh of each participant, between the knee and the trunk, using an adhesive strip. Participants will use the devices for 7 days at each follow-up visit (baseline, 3, and 12 months). This device is used to estimate the average minutes of activity per day, average minutes spent sitting per day, and average number of steps per day. We will use cut-off points for the categorization of the sedentary behavior (0–5 counts/15 s) collected through the ActivPal devices, as proposed by Atkin et al.^[50]^

#### 2.6.4 Caloric intake

Caloric intake (kcal/d) and dietary habits of participants will be evaluated using a semi-quantitative food-frequency questionnaire previously validated in Spain.^[51]^ The estimated frequency corresponds to the previous year at the time of the interview and is based on the typical portion sizes multiplied by the consumption frequency for each food. This value is then divided into 9 intake categories ranging from never to >6 servings/d. This will be used to estimate daily intake of macro- and micro-nutrients. Adherence to the Mediterranean diet will be assessed using the validated 14-point Mediterranean Diet Adherence Screener (MEDAS),^[52]^ developed by the PREDIMED study group, which explores compliance with various aspects considered characteristic of the Mediterranean diet. Adequate adherence will be assumed when the total score is 9 points or higher.

#### 2.6.5 Quality of life

Quality of life will be measured using the IWQoL-Lite (Impact of Weight on Quality of Life-Lite). This questionnaire is made up of 31 self-reported items, each of which is scored between 1 (“never true”) and 5 (“always true”). Specific scores are obtained in 5 domains: physical function (11 items), self-esteem (7 items), sexual life (4 items), public distress (5 items), and work (4 items). Higher scores are associated with better quality of life. The original version proved to have good internal consistency (≥0.90), test–retest reliability (≥0.83), and sensitivity to change.^[53]^

#### 2.6.6 Arterial aging

The central and peripheral augmentation index will be measured using a novel wrist-worn device developed by Microsoft Research. Participants will be examined in a seated position after 10 minutes of rest. The subject's arm will be supported on a firm surface at heart-height. The wrist-worn device non-invasively senses multiple physiological signals associated with cardiovascular health. The device includes an applanation tonometer placed over the radial artery, an electrocardiogram, a 3-axis accelerometer, and a 3-axis gyroscope. It is capable of performing continuous or scheduled measurements using various configurations of these sensors and it includes Bluetooth for data streaming.^[54]^ Cardio-ankle vascular index (CAVI), brachial-ankle pulse wave velocity (ba-PWV), and ankle-brachial index (ABI) will be measured at rest, using the Vasera device VS-2000 (Fukuda Denshi).

#### 2.6.7 Pro-Inflammatory markers

A centralized laboratory will measure levels of IL-1β, IL-7, and adiponectin, as they are considered pro-inflammatory markers related to obesity.^[55]^ Venous blood sampling will be performed between 8:00 am and 9:00 am after participants have fasted and abstained from smoking, consumption of alcohol, and caffeinated beverages for 12 hours. Other laboratory determinations, such as plasma glucose, creatinine, total serum cholesterol, high-density lipoprotein (HDL) cholesterol, low-density lipoprotein (LDL) cholesterol, and triglyceride concentrations will be determined using standard automated enzymatic methods. Glycosylated hemoglobin, high-sensitivity C-reactive protein levels, insulinemia, and fibrinogen values will be measured with an immune-turbidimetric assay. An ELISA will be used to determine the levels of thyroid-stimulating hormone (TSH) and free T4 hormone. Spot morning urine samples will be collected to determine the albumin–creatinine ratio. The analyses will be performed at the hospitals in the cities participating in external quality assurance programs of the Spanish Society of Clinical Chemistry and Molecular Pathology.

#### 2.6.8 Other variables

Sociodemographic variables: At the time of inclusion in the study, data on age, sex, marital status, educational level, and occupation will be collected.

Peripheral blood pressure: Three measurements of systolic (SBP) and diastolic (DBP) blood pressure will be performed using the average of the last 2 with a validated Omron M10-IT model sphygmomanometer (Omron Healthcare, Kyoto, Japan). The measurements will be made on the participant's dominant arm in a seated position after at least 5 minutes of rest with a cuff of appropriate size, as determined by measurement of the upper arm circumference and following the recommendations of the European Society of Hypertension.^[56]^

Smoking status: This will be assessed through a questionnaire of 4 standard questions adapted from the WHO MONICA study.^[57]^ Study participants will be classified as current smokers or non-smokers (never or >1 year without smoking).

Motivation to change: We will use the motivation stage according to the transtheoretical model of change (TTM)—Prochaska and DiClemente model (pre-contemplation, contemplation, preparation, action, maintenance, and relapse).^[58]^ TTM constructs will be measured only when relating to weight loss.

### 2.7 Intervention

#### 2.7.1 Standard counselling (CG and IG)

Both groups (control and intervention) will receive 5 minutes of counselling at the end of the baseline visit and prior to randomization. Advice on physical activity will be in compliance with the current international recommendations for the general population. The health benefits of physical activity and the recommendation to complete at least 30 minutes of moderate activity 5 days a week, or 20 minutes of vigorous activity 3 days a week, will be explained. Counselling on food will be in compliance with the plate method,^[59]^ in which a plate is divided into 4 parts: half the plate for salad or vegetables, one fourth for proteins (white meat preferred over red meat), and the final fourth for carbohydrates. In addition, a medium-sized piece of fruit and a skimmed dairy product should be consumed for dessert.

#### 2.7.2 Specific intervention (IG)

**Figure 2:**
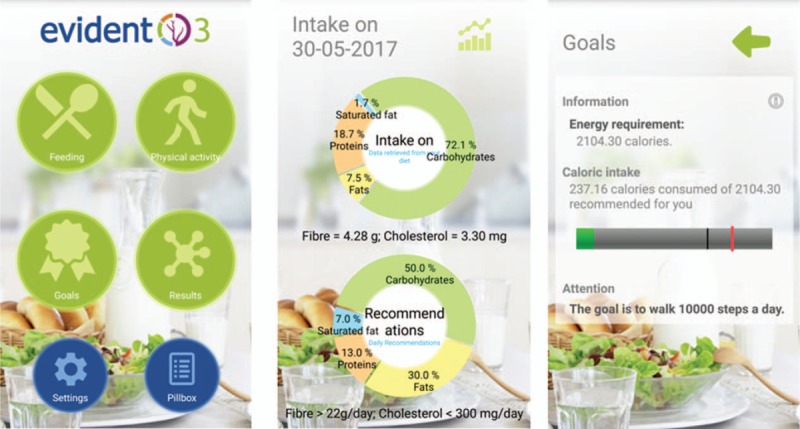
Screenshots of the EVIDENT 3 application.

The IG will be lent a smartphone and a smartband (Xiaomi Miband 2), for a 3-month period, corresponding to the length of the intervention. A baseline visit will be completed, lasting approximately 15 minutes, in which the subjects will be trained to use the device and the application (EVIDENT 3 Application, [record entry no. 00/2017/2438]) specifically designed for the study (Fig. 2). In the first part of this visit, the application will be configured with each participant's data (sex, age, weight, and height). The application design allows for full daily control of food intake and physical activity performed (more details in Supplementary Material). The application collects the information gathered by the smartband on physical activity, calories, number of steps, number of hours walking, and the information gathered by the subject on daily food intake. Once all the daily information is collected, the application integrates the data to create, based on the subject's characteristics, personalized recommendations, and specific objectives and goals for weight loss. A new visit will take place the week the device is provided, to confirm it is being used properly and to answer any questions. At the 3-month visit, the devices will be collected. All information generated by the application will be duly analyzed and entered in the database. We will evaluate the usability and acceptability of the application^[60]^ after completing the 3-month assessment visit only for IG subjects. A questionnaire will be used to evaluate the application's ease of use, need for training by expert personnel, level of complexity, and its consistency.

#### 2.7.3 Blinding strategy

The investigator carrying out the intervention with the IG will be different from the person responsible for the assessment and the standard counselling; both will be kept blinded throughout the study, as will the investigator conducting the data analysis. The subjects, due to the nature of the study, cannot be blinded. To prevent contamination between groups, in the assessment visits (3 and 6 months), only the study variables will be evaluated but no advice or reinforcement will be given. In addition, the application will not be available for download on the web until the end of the study so the CG will not be able to make use of it in any way. During the follow-up visits, participants will be instructed not to use other health technologies. Compliance will be ensured by a brief question about the use of other applications and mHealth technology.

### 2.8 Statistical analysis

#### 2.8.1 General analysis

Results will be expressed as mean ± standard deviation for quantitative variables normally distributed or medians (interquartile ranges) for the skewed variables and by frequency distribution for qualitative variables. The statistical normality will be tested using the Kolmogorov–Smirnov test. The Chi-square test and Fisher exact test will be used to analyze the association between independent qualitative variables. The Student's *t* test or Mann–Whitney *U* test will be used for the comparison of means between 2 independent groups. Pearson correlation or Rho de Spearman will be used to evaluate the relationships between quantitative variables.

The main analysis of primary and secondary outcomes will be performed on an intent-to-treat basis. We will also perform a secondary analysis according to the different levels of adherence to the use of the application (low adherence: <2 months; high adherence: ≥2 months) and according to relevant subgroups. Multiple imputations will be used to address missing values in the dataset.

### 2.9 Analysis of intervention effect in primary and secondary outcomes

To analyze the changes, at 3 and 12 months from baseline, in primary (weight loss) and secondary endpoints within the same group, we will use the paired *t* test or McNemar test for quantitative or dichotomy variables, respectively.

To analyze the effect of the intervention, we will compare the changes from baseline observed between the IG and CG in weight (primary endpoint) and secondary endpoints, using ANCOVA at 3 and 12 months, adjusting for baseline measures of each variable.

In order to analyze the group effect in the follow-up for primary and secondary endpoints, we will perform a multivariate analysis of the variance of repeated measures, adjusted by the baseline value of each variable. Any interaction between the primary and secondary endpoints with the group (IG vs CG) and Cohen *d* will be estimated.

### 2.10 Analysis of effect by adherence

To assess the effect of adherence to the application, we will divide the IG into high adherence (≥2 months), and low adherence (<2 months), according to the specified criteria, and the same analysis as previously described will be performed.

### 2.11 Analysis by subgroups

The impact of the intervention could be also affected by age, cultural and socioeconomic level, and certain diseases, which will all be analyzed by specific subgroups as previously described. An analysis will be performed to assess differences between men and women for adherence to the application and results in the short term (3 months) and long term (12 months).

### 2.12 Secondary analysis

A multivariate analysis of multiple linear regressions will be performed to identify the variables that most influence changes in weight, physical activity, sedentarism, and arterial stiffness.

Continuous information, nutrition, and physical activity, over the 3 months of use of the application and the Smartband, will be collected in the IG. Data will be grouped by week to observe their evolution and compare the changes in these parameters with changes in the main variables (body weight, physical activity, and arterial stiffness).

All statistical analyses will be performed with SPSS version 23.0 (IBM Corp, Armonk, NY), with the level of statistical significance set at *P* ≤.05.

### 2.13 Ethics and dissemination

The study was approved by the Clinical Research Ethics Committee of the Health Area of Salamanca (“CREC of Health Area of Salamanca”) in April 2016. In addition, the study was approved by the Ethics Committees of the 4 collaborating centres (CEIC de Aragón [CEICA], CEIC de Castilla la Mancha, CEIC de Baleares and CEIC de Area de Salud de Valladolid Oeste). A SPIRIT checklist is available for this protocol. The trial has been registered in ClinicalTrials.gov with identifier NCT03175614.

Participants must sign the informed consent before inclusion in the study in accordance with the Declaration of Helsinki. Subjects will be informed about the objectives of the study and the risks and benefits of the examinations that they will undergo, including sample collection. None of the testing could result in life-threatening risks for the subjects. Subject confidentiality will be ensured at all times in accordance with current laws and regulations on personal data protection (LOPD 15/1999 of 13 December 1999) as well as the conditions described in Act 14/2007 on biomedical research.

## 3 Discussion

The studies available that quantify the effect of smartphone applications (mHealth) on weight loss are increasing. There is a great diversity in the applications used obtaining different results on lifestyle improvement. These mHealth interventions have small to moderate effects on physical activity and sedentary behavior.^[61]^ Recent evidence suggests that applications must include a detailed registration of foods, goals, and customizable challenges and also incorporate personalized feedback and recommendations.^[62]^ Finally, the populations studied are not homogeneous, and therefore generate different results depending on age, sex, and morbidities. Therefore, it is expected that the results of this study will further our understanding of the efficacy of new technologies, combined with traditional counselling, towards reducing obesity and enabling healthier lifestyles.

### 3.1 Methodological limitations

Because of the nature of the intervention, participating subjects cannot be blinded. However, towards achieving the greatest possible blinding effect, neither the person performing the statistical analysis nor the person measuring study variables will know to which group each participant belongs. Even though the analysis of some of the results regarding the modification of lifestyle will be performed using self-reported data, validated tools will be used. However, objective data, such as accelerometer for physical activity and weight and body composition measurement, will compliment subjective results. Any difficulties involved in the use of the application may result in an increase in the number of dropouts from the IG.

### 3.2 Dissemination plan

We will use a variety of methods to ensure that our work will achieve maximum visibility. The publication of our study protocol provides an important first step in this direction. In this paper, we have sought to offer a comprehensive overview of relevant literature while underlining current research gaps that necessitated the design and implementation of the EVIDENT 3 study. The study results, given their applicability and implications for the general population, will be disseminated in investigator meetings and in articles published in peer-reviewed scientific journals. Minimally, one manuscript is planned for publication with main findings and sub-analyses (in Supplementary Material) and one manuscript for the secondary findings. In addition, a proposal for a doctoral thesis based on this project has been made.

## Acknowledgments

The authors are grateful to all the professionals participating in the EVIDENT 3 study:

Centro de Salud La Alamedilla de Salamanca: Luis Garcia-Ortiz, Jose I. Recio-Rodriguez, Cristina Agudo-Conde, Manuel A. Gomez-Marcos, Rosario Alonso-Dominguez, Natalia Sanchez-Aguadero, Angela de Cabo-Laso, Carmela Rodriguez-Martin, Carmen Castaño-Sanchez, Benigna Sanchez-Salgado, Emiliano Rodriguez-Sanchez, Susana Gonzalez-Sanchez, Jesus Gonzalez-Sanchez, Maria C. Patino-Alonso, Jose A. Maderuelo-Fernandez, Rafael Hipola-Muñoz, Leticia Gomez-Sanchez, Cristina Martin-Martin, Cristina Lugones-Sánchez.

Centro de Salud Torreramona de Zaragoza: Natividad González-Viejo, José Félix Magdalena-Belio, Luis Otegui-Ilarduya, Francisco J. Rubio-Galan, Cristina I. Sauras-Yera, Amor Melguizo-Bejar, Maria J. Gil-Train, Marta Iribarne-Ferrer, Olga Magdalena-González, Miguel A. Lafuente-Ripolles.

Centro de Salud Cuenca I: Fernando Salcedo-Aguilar, Fructuoso Muelas-Herraiz, Maria A. Molina-Morate, Amparo Pérez-Parra, Fernando Madero, Angel Garcia-Imbroda, Jose M. Izquierdo, María L. Monterde, Alba Soriano-Cano.

Centro de Salud Sta Ponça de Palma de Mallorca: José I. Ramírez-Manent, José L. Ferrer-Perelló, José E. Romero-Palmer, Manuel Sarmiento-Cruz, Guillermo Artigues, Sebastian March, María Albaladejo-Blanco, Margarita I. Moyá-Seguí, Cristina Vidal-Ribas, Patricia Lorente-Montalvo, Isabel Torrens-Darder Maria M. Torrens-Darder, Lucia Pascual.

Centro de Salud San Pablo de Valladolid: Maria J. Álvarez-Miguel, Maria D. de Arriba-Gómez, Maria Á Rodríguez-Fernández, Isabel Arranz-Hernando, Silvia Ramos-de la Torre, Amparo Arqueaga-Luengo, Maria E. Moreno-Moreno, Agustina Marcos-García, Nora Manrique-Vinagre, Nieves Palomo-Blazquez, José L. Montalvillo-Montalvillo, Maria E. Fernández-Rodríguez, Alejandro González-Moro, Marta Santiago-Pastor, Maria I Pérez-Concejo, Aurora Rubio-Fernández.

Centro de Salud Casa del Barco de Valladolid: Amparo Gomez-Arranz, Carmen Fernandez-Alonso, Daniel Rodriguez-Dominguez, Irene Repiso-Gento, Aventina de la Cal-de la Fuente, Rosa Aragon-Garcia, Miguel A. Diez-Garcia, Elisa Ibañes-Jalon, Ines Castrillo-Sanz, Ana M. Corcho-Castaño, Esther Jimenez-Lopez, Daniel Correa-Gonzalez, Lucia Barruso-Villafaina, Isabel Peña-Garcia, Dolores Escudero-Terron, Pilar Mena-Martin, Rosario Fraile-Gomez, Alberto Alonso-Gomez, Pilar Urueña, Francisca Martinez-Bermejo, Concepción Hernandez-San Jose, Manuela Nuñez-Gomez, Patricia Sanz-Capdepont, Ana I Pazos-Revuelta, Sofia Perez-Niño, María E. Junquera-del Pozo.

CGB Computer Company, Salamanca, Spain, contributed to the technical development of EVIDENT 3 application.

## Supplementary Material

Supplemental Digital Content
