# Supplementary material for: EVIDENT 3 Study: A randomized, controlled clinical trial to reduce inactivity and caloric intake in sedentary and overweight or obese people using a smartphone application: Study protocol
Source: Medicine (Baltimore). 2018 Jan 12;97(2):e9633. doi: 10.1097/MD.0000000000009633 (PMC5943855; doi:10.1097/MD.0000000000009633)
Supplement: Supplemental Digital Content [file medi-97-e9633-s001.docx]

Supplementary material:

TECHNICAL REPORT OF THE SMARTPHONE APPLICATION

Software Product Lines (SPL) combine systematic development and reuse of coarse-grained components that include the common and variable parts of the product. It is proposed to use the standard UML package mix relation to represent an SPL architecture with conventional variations and tools, as well as some of the most widespread and consolidated design patterns within the software development industry such as MVC, BO, DAO and VO, thus achieving great advantages such as reuse of components, easy structuring of the application, greater scalability, easier testing, or easier future maintenance of the application. The product is initially intended as a development for Android devices, so we will use the Java, Eclipse and Android SDK platforms, always applying the precepts above. There will be a differentiation of the presentation layer (where we will have XML files and different resources that will define the views that our application will use) and its core, where its functionality will reside. This functionality will be fully implemented in Java and packaged. In each package we will collect all those classes that are placed under the same module or framework of functionality, thus having all the elements of our code that model a common characteristic well differentiated. Thinking about the possible internationalization of the application and thus its easy translation in the future, the resources of both text and images will be separated by folders that follow the same nomenclature, assigning each of them their language through its corresponding abbreviation According to ISO 693-1. Thus, depending on the language that the terminal has configured, the application will feed on the elements defined for that language automatically, without having to configure it explicitly.

As for the application modules, the following are established: Connection and access to the database management system: the application data is stored in a local SQLite database, especially appropriate for the technology used, since It is a small size system that does not require configuration and is also available on all terminals with Android operating system. The design patterns DAO and VO will be used. Thanks to this, we will encapsulate access to the database for later use in the business logic layer.

The calculation of physical activity will be done through a smartband, which is a device that is worn on the wrist and complements the functionality of the application. With this smartband, the data of daily activity performed by the user (in the same way as with the pedometer) is collected, but other important data such as the heart rate of the user are also recorded. This device will provide different data that are collected by the application through a service, component used for long-term operations in the background, making it independent of the rest of the application (where the user will interact with it). Upon their collection, these data are processed in the application and from the conclusions that can be extracted from them the user would be notified in one way or another, depending on the urgency and obligatory nature of the notice or notification.

Notifications: thanks to the data sampled, both from the ones manually inserted by the user, and from the data collected from the smartband, and that have been stored in the database, customized calculations will be made, depending on the physical characteristics.

With these calculations, different messages will be displayed programmed daily or weekly, indicating to the user what have been their dietary and physical patterns, in addition to different recommendations. In order to allow timely planning of these messages, we will use Broadcast Receivers, Android components that are registered for the detection of certain events (in our case, a certain day and time), so the application will be invoked as planned, showing the notification that has been assigned. As a link between the different parts, we will have a main module, which houses the different activities of the application. Within this module, we can group the functionality into four parts: -Diet: where data are collected from the user's daily intake. - Physical activity: here the user will introduce the different activities that he does throughout the day, as long as they are not already included in the calculations made by the pedometer. -Calendar: serves as a link between the various options of the application, but differentiating them over time. -Configuration-Administration: where you define the user data and different parameters that will later influence the calculations.
